# P-Selectin Glycoprotein Ligand-1 Forms Dimeric Interactions with E-Selectin but Monomeric Interactions with L-Selectin on Cell Surfaces

**DOI:** 10.1371/journal.pone.0057202

**Published:** 2013-02-25

**Authors:** Yan Zhang, Ning Jiang, Veronika I. Zarnitsyna, Arkadiusz G. Klopocki, Rodger P. McEver, Cheng Zhu

**Affiliations:** 1 Woodruff School of Mechanical Engineering, Georgia Institute of Technology, Atlanta, Georgia, United States of America; 2 Coulter Department of Biomedical Engineering, Georgia Institute of Technology, Atlanta, Georgia, United States of America; 3 Cardiovascular Biology Research Program, Oklahoma Medical Research Foundation, University of Oklahoma Health Sciences Center, Oklahoma City, Oklahoma, United States of America; 4 Department of Biochemistry & Molecular Biology, University of Oklahoma Health Sciences Center, Oklahoma City, Oklahoma, United States of America; Brigham and Women’s Hospital, United States of America

## Abstract

Interactions of selectins with cell surface glycoconjugates mediate the first step of the adhesion and signaling cascade that recruits circulating leukocytes to sites of infection or injury. P-selectin dimerizes on the surface of endothelial cells and forms dimeric bonds with P-selectin glycoprotein ligand-1 (PSGL-1), a homodimeric sialomucin on leukocytes. It is not known whether leukocyte L-selectin or endothelial cell E-selectin are monomeric or oligomeric. Here we used the micropipette technique to analyze two-dimensional binding of monomeric or dimeric L- and E-selectin with monomeric or dimeric PSGL-1. Adhesion frequency analysis demonstrated that E-selectin on human aortic endothelial cells supported dimeric interactions with dimeric PSGL-1 and monomeric interactions with monomeric PSGL-1. In contrast, L-selectin on human neutrophils supported monomeric interactions with dimeric or monomeric PSGL-1. Our work provides a new method to analyze oligomeric cross-junctional molecular binding at the interface of two interacting cells.

## Introduction

During acute inﬂammation, leukocytes are recruited from the circulation to sites of infection and injury. This multistep adhesion and signaling cascade is initiated by interactions between selectins and their glycoconjugates that mediate leukocyte tethering to and rolling on the surface of endothelial cells [1]. The selectin family includes three members. L-selectin is expressed on leukocytes. P- and E-selectin are expressed on activated platelets and/or activated endothelial cells. Their common structure includes a ligand-binding lectin domain, an epidermal growth factor-like module, multiple copies of consensus repeats characteristic of complement-binding proteins, a transmembrane segment, and a short cytoplasmic domain [1]–[3]. The best characterized selectin ligand is P-selectin glycoprotein ligand-1 (PSGL-1), a high-affinity ligand for P-selectin that also binds to L- and E-selectin [1]–[3].

P-selectin [4], [5] and PSGL-1 [6]–[8] form dimers on the respective surfaces of endothelial cells and leukocytes. Dimerization of P-selectin and PSGL-1 stabilizes cell rolling and enhances tether strength in shear flow [9] and prolongs bond lifetime [10]. Induced cell-surface dimerization of L-selectin by cross-linking its cytoplasmic or extracellular domains increases binding to soluble ligand mimic and adhesion to immobilized ligand or to vascular endothelium under shear [11], [12]. However, biochemical analyses of the L-selectin transmembrane and cytoplasmic domains suggest that they are monomeric in bacterial or synthetic membranes [13]. PSGL-1 does not form dimeric bonds with E-selectin purified from lysates of CHO cell transfectants after it is reconstituted into glass-supported lipid bilayers [14]. However, it is not known whether E-selectin forms monomers or oligomers on the surfaces of endothelial cells.

Like other cell adhesion receptors, selectins and their ligands also transduce signals [1], [15]. Antibody-mediated crosslinking of L-selectin or PSGL-1 on leukocytes or of E- or P-selectin on endothelial cells triggers intracellular calcium fluxes and protein tyrosine phosphorylation. Receptor tyrosine kinases [16] and multichain immune recognition receptors [17] must form dimers to transduce signals. On the other hand, both dimeric and monomeric forms of PSGL-1 transduce signals after engaging P-selectin [18]. Whether other selectin ligands or selectins themselves must dimerize to signal under physiological conditions is not known.

The goal of the present work was to determine whether L- and E-selectin form oligomers or monomers on cell surfaces. We used the micropipette adhesion frequency assay to analyze two-dimensional (2D) binding between selectins and ligands [19]–[22]. Our strategy was to use purified molecular systems with structurally well-defined multimericities to define the requirements and properties of monomeric and dimeric interactions between the same receptor-ligand pair. By comparing binding to the same densities of native dimeric PSGL-1 and recombinant monomeric soluble (s) PSGL-1 [10], we showed that endothelial cell E-selectin supported dimeric interactions whereas neutrophil L-selectin supported monomeric interactions.

## Materials and Methods

### Cells and Proteins

Chinese hamster ovary (CHO) cells transfected to stably express human E-selectin were previously described [23]. Human aortic endothelial cells (HAECs, Clonetics, Walkersville, MD) were generous gifts from Dr. Julia Babensee (Georgia Institute of Technology) and were maintained as previously described [24]. CHO cells were cultured in RPMI 1640 plus 5% fetal bovine serum, 5% calf serum, 2 mM glutamine, 1 mM sodium pyruvate, and 1% penicillin/streptomycin. To induce expression of E-selectin, HAECs were stimulated with 100 U/ml interleukin-1β (IL-1β) for 6 h.

Neutrophils were isolated from a drop of whole blood via a finger prick and RBCs were isolated from peripheral blood drawn from healthy volunteers according to protocols approved by Georgia Institute of Technology’s Institutional Review Board [25]. Written informed consent were obtained for all volunteers. Neutrophils were prepared on the day of experiment. After RBCs were lysed by a brief hypotonic shock, neutrophils were spun down and resuspended in Hanks’ balanced salt solution (Sigma-Aldrich, St. Louis, MO) with 1% human serum albumin (ZLB Plasma, Boca Raton, FL), and used immediately. RBCs were prepared and stored for experiments in several weeks. Whole blood was layered over Histopaque 1119 (Sigma) and centrifuged by 2000 g for 30 min at room temperature. The pelleted RBCs were washed, coated with capturing antibodies, and stored in experimental additive solution 45 (EAS 45) (Dumaswala et al., 1996) at 4°C.

Human L-selectin-Ig was expressed and purified as previously described [26]. The same methods were used to construct, express, and purify human E-selectin-Ig, comprising the lectin and epidermal growth factor domains and the first two consensus repeats of human E-selectin fused to the Fc portion of human IgG1. Recombinant human sE-selectin [27], sPSGL-1 [28], membrane PSGL-1 [8], anti-E-selectin capturing and blocking monoclonal antibodies (mAbs) 1D6 [29] and ES1 [23], anti-L-selectin mAb DREG56 [30], and anti-PSGL-1 capturing mAb PL2 [31] have been described previously. Goat anti-human IgG antibody (for capturing selectin-Ig) and FITC-labeled goat anti-mouse IgG mAb A85-1 (used as secondary antibody) or FITC-labeled human IgG antibody (for site density measurement) was from Sigma. PE-labeled anti-PSGL-1 mAb PL1 for site density measurement was from Calbiochem (St. Louis, MO).

### Coupling Ligands onto RBC Surface

A previously described modified chromium chloride (CrCl_3_) method [32] was used to couple capturing antibodies (1D6 for sE-selectin, goat anti-human IgG-Fc for L- and E-selectin-Ig, and PL2 for (s)PSGL-1) on RBC. Briefly, a 1% CrCl_3_ solution was prepared, aged at pH 5, and diluted in 20 mM acetate-buffered saline, pH 5.5, at ratios ranging from 1∶100 to 1∶2400. RBCs were washed five times in saline and resuspended in 1% hematocrit. Antibodies were added to each 250 µl sample and mixed. An equal volume of diluted CrCl_3_ solution was added dropwise with constant agitation. After 5 min the reaction was stopped by addition of 0.5 ml phosphate buffered saline plus 1% IgG-free bovine serum albumin (BSA). Aliquots from each sample were examined under light microscopy for aggregation. Cells were subsequently washed and stored in EAS45. Immediately prior to the micropipette experiment, RBCs precoated with 1D6, anti-Fc or PL2 were respectively incubated with sE-selectin (0.5 µg/ml), L- or E-selectin-Ig (0.5 µg/ml), or (s)PSGL-1 (0.2 µg/ml) at room temperature for 30 min.

### Micropipette Adhesion Frequency Assay

**Figure 1:**
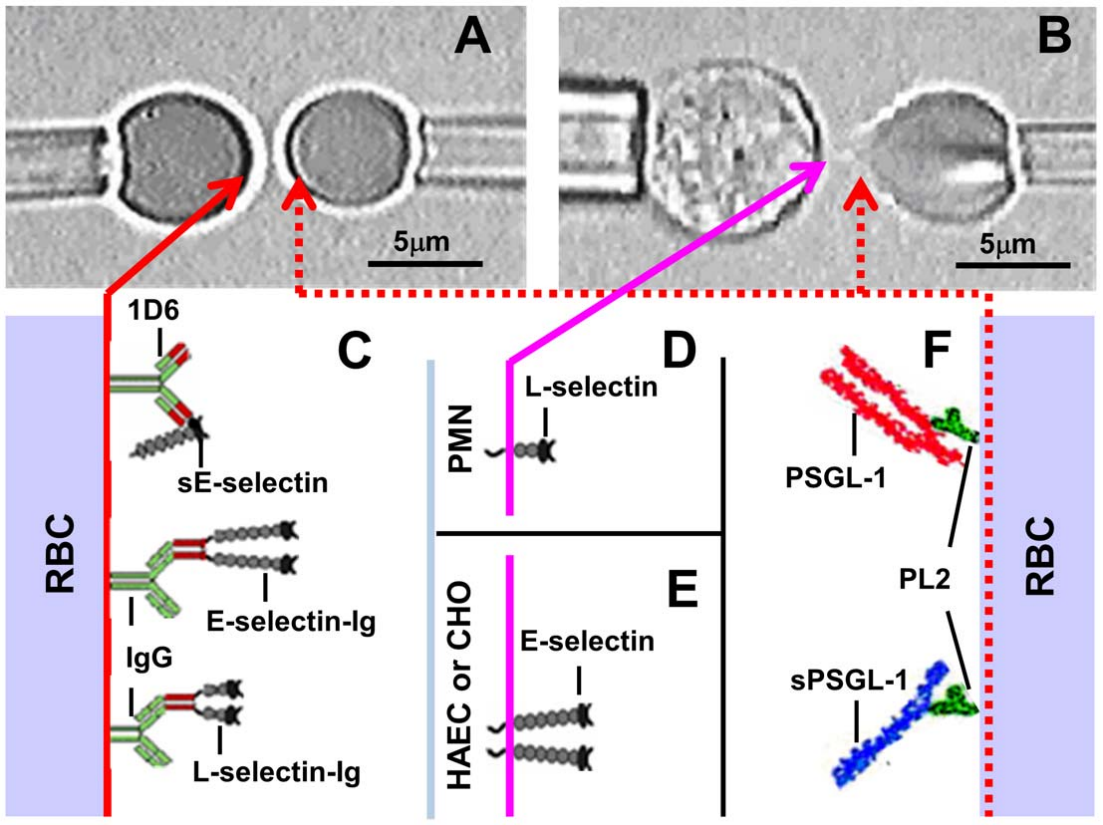
Micropipette adhesion frequency assay. A and B . Photomicrographs of a pair of RBCs (A) or a nucleated cell (left) and a RBC (right) (B) respectively held by two apposing pipettes. C-F. Composite of interacting molecules on respective cell surfaces. The left RBC in A was precoated with anti-E-selectin (1D6) that captured monomeric sE-selectin or precoated with goat anti-human Ig that captured dimeric E-selectin-Ig or L-selectin-Ig (C). The left nucleated cell in B was a PMN that expressed L-selectin (D) but could also be a HAEC or CHO cell that expressed E-selectin (E). Based on the data of this work, L-selectin on PMN is depicted as a monomer whereas E-selectin on HAEC and CHO cell is depicted as a dimer. The right RBCs in both A and B were precoated with anti-PSGL-1 mAb PL2 that captured dimeric membrane PSGL-1 or monomeric recombinant sPSGL-1 (F).

A previously described micropipette adhesion frequency assay [19]–[22] was used to analyze the two-dimensional (2D) binding between various forms of L- or E-selectin and (s)PSGL-1. Briefly, a cell chamber filled with 3 ml of L-15 medium supplemented with 1% BSA was mounted on the stage of an inverted microscope (Zeiss Axiovert 100, Oberkochan, Germany). Cells coated with selectins or ligands were added to different locations separated by sufficient distance to avoid mixing. A selectin-coated cell was picked up by one micropipette and a ligand-coated cells was picked up by an apposing micropipette. The cells were then aligned via micromanipulation. Selectins were either captured on RBCs by precoated antibodies (Fig. 1C), expressed on human neutrophils (for L-selectin, Fig. 1D), or expressed on CHO cell transfectants or induced on HAECs (for E-selectin, Fig. 1E). (s)PSGL-1 was captured on RBCs by precoated mAb (Fig. 1F). One pipette was driven by a computer-controlled piezo translator to move the cell to contact the other cell held stationary for a pre-determined area and duration. Following pipette retraction, the cells were either immediately separated (no adhesion, scored 0) or remained bound with the ﬂexible RBC(s) being stretched (adhesion, scored 1) for a short time until detachment. The likelihood of adhesion was estimated from the frequency of adhesions (*P*_a_) observed in 50∼100 repeated contact cycles using a single pair of cells. Three to five cell pairs were tested to obtain a mean *P*_a_ ± S.E.M. for that contact duration (*t*). Measurements were made in five contact durations to obtain a *P*_a_ vs. *t* binding curve for each receptor-ligand pair respectively expressed on the corresponding cells at given site densities measured separately by flow cytometry.

### Site Density Determination

Cell samples were incubated with saturating concentrations of fluorescence-conjugated primary antibodies (10 µg/ml or according to manufacturer’s instruction) in FACS solution (RPMI with 1% BSA and 0.05% sodium azide) for 30 min followed by three washes, and analyzed immediately. If non-conjugated primary antibody was used, cells were next incubated with FITC-conjugated secondary antibody (10 µg/ml or per manufacturer’s instruction) and then washed and analyzed immediately. Cells and calibration beads from quantum FITC MESF kits (Bangs Laboratories, Fishers, IN) were analyzed on a BD LSR II flow cytometer (Becton-Dickinson Immunocytometry Systems, San Jose, CA). The mean fluorescent intensities (MFIs) were recorded for samples as well as for each peak of the calibration beads. A standard curve was plotted using the MFIs of the calibration beads, which was used to calculate molecules of equivalent soluble fluorophore (MESF) from the MFIs measured from the cells. Antibodies bound per cell were then calculated by dividing MESF by fluorophore per antibody (supplied by manufacturers), which were then converted to site densities by dividing by the cell surface area [33], [34].

The capacity of capturing E- or L-selectin-Ig by goat anti-human IgG antibody precoated on RBCs was measured using a FITC-conjugated human IgG. At saturating concentration, each binding site for Fc should capture one dimeric E- or L-selectin-Ig. Direct measurement using anti-E-selectin (ES1) and anti-L-selectin (DREG56) mAbs obtained similar results. The site density of sE-selectin was controlled by the site density of its capture antibody (1D6) precoated on RBCs, which was measured using FITC-labeled goat anti-mouse IgG mAb A85-1. The site density of sPSGL-1 was measured using PE-labeled anti-PSGL-1 mAb PL1. Since the same batch of RBCs pre-coated with the same density of PL2 were incubated with saturating concentrations of PSGL-1 or sPSGL-1, only one leg of PSGL-1 would be captured by PL2. Thus, the site density for dimeric PSGL-1 was inferred to be the same as that of monomeric sPSGL-1, whereas the density of selectin binding-sites of PSGL-1 should be twice as that of sPSGL-1. It is possible that a fraction of PL2 bound dimerically with the dimeric PSGL-1 even though a saturating concentration was used. Should this be the case, using our indirect inference would overestimate the selectin binding sites on PSGL-1-coated RBCs. This would increase, rather than decrease, the confidence of our interpretation regarding the formation of dimeric bonds from the observation of higher selectin binding to PSGL-1-coated RBCs than to sPSGL-1-coated RBCs. The site densities of E-selectin on CHO cells and HAECs and of L-selectin on neutrophils were measured using anti-E-selectin mAb ES1 and anti-L-selectin mAb DREG5.

## Results

To discriminate the multimericities of E- and L-selectin on their respective cell surfaces, we used the micropipette adhesion frequency assay, which measures cross-junctional ligand interaction with a cell surface receptor in its native membrane environment [21], [33], [35]. In this 2D assay, the measured adhesion frequency depends on both densities of the receptor and the ligand. To control for this, we used the same batch of cells bearing the same receptor density to interact with RBCs coated with the same density of PL2 to capture dimeric (PSGL-1) or monomeric (sPSGL-1). This was done for both L-selectin as well as E-selectin. Using this *in situ* kinetic analysis, we determined the ability for endothelial cell E-selectin and neutrophil L-selectin to support dimeric interactions by comparing their binding properties with PSGL-1 and sPSGL-1.

### Interactions of sPSGL-1 and PSGL-1 with sE-selectin were Indistinguishable Whereas those with E-selectin-Ig were Distinct

**Figure 2:**
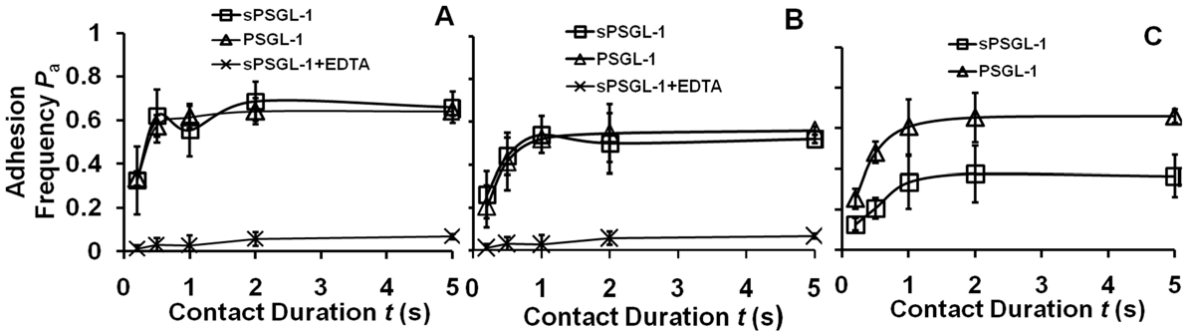
Binding curves of two forms of recombinant E-selectin interacting with (s)PSGL-1. Adhesion frequency vs. contact duration plots of RBCs coated with sE-selectin at site density of 126 µm^−2^ (A) or 46 µm^−2^ (B) or E-selectin-Ig at site density of 124 µm^−2^ (C) interacting with RBCs coated with sPSGL-1 (square) or PSGL-1 (triangle) at matched densities. Control for nonspecific adhesion was measured using 6 mM EDTA to inhibit binding between sE-selectin and sPSGL-1 (A and B, cross). The different adhesion frequency levels of sE-selectin–PSGL-1 (A) and E-selectin-Ig–sPSGL-1 (C) interactions may result from the differences in how the recombinant proteins were made, how sE-selectin and E-selectin-Ig were coated on RBCs, and how their site densities were measured.

We measured adhesion frequency *P*_a_ vs. contact duration *t* curves for RBCs coated with matched densities of either sPSGL-1 or PSGL-1 and RBCs coated with sE-selectin at two site densities (Fig. 2A and B) or with E-selectin-Ig (Fig. 2C). In contact durations from 0.25–5 s, *P*_a_ increased with *t* initially and then reached a plateau, as previously observed for E-selectin interaction with carbohydrate ligands on HL-60 cells and Colo-205 cells [36]. Nonspecific adhesion was controlled by adding EDTA to chelate calcium, a divalent cation required for selectin-ligand binding.

It is evident that the two curves of (s)PSGL-1 binding to sE-selectin were indistinguishable regardless of the sE-selectin site densities used (Fig. 2, A and B). This indicates that monomeric sE-selectin formed the same type of monomeric bonds with both monomeric sPSGL-1 and dimeric PSGL-1. It also suggests that each of the two members of dimeric PSGL-1 interacted with sE-selectin with the same binding properties. In sharp contrast, the two curves of (s)PSGL-1 binding to E-selectin-Ig were distinctly different, with higher binding for the dimeric PSGL-1 than for the monomeric sPSGL-1 at all contact durations (Fig. 2C). This indicates that dimeric E-selectin-Ig formed monomeric bonds with monomeric sPSGL-1 but dimeric bonds with dimeric PSGL-1.

### Interactions of (s)PSGL-1 with E-selectin on HAECs and CHO Cells Resembled those with E-selectin-Ig on RBCs

**Figure 3:**
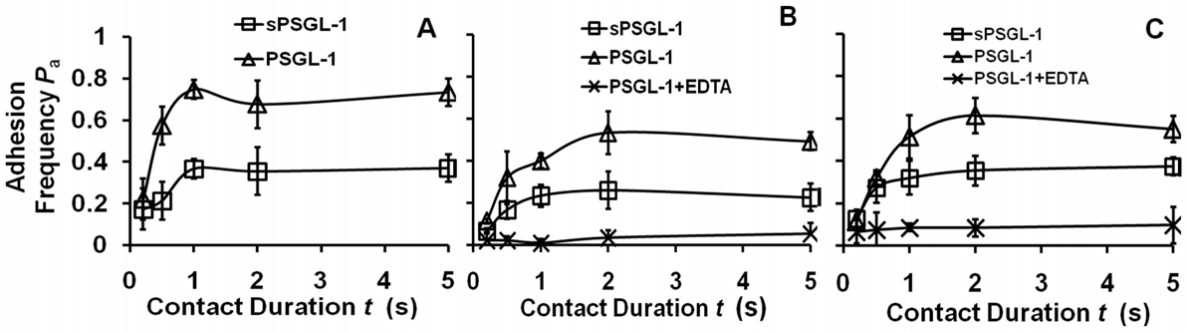
Binding curves of cell surface E-selectin interacting with (s)PSGL-1. Adhesion frequency vs. contact duration plots of HAEC induced to express native E-selectin at 419 µm^−2^ (A) or 258 µm^−2^ (B) or CHO cells transfected to express recombinant E-selectin at 410 µm^−2^ (C) interacting with RBCs coated with matched molecular densities of sPSGL-1 (square) or PSGL-1 (triangle). Control for nonspecific adhesion was measured using 6 mM EDTA to inhibit binding between E-selectin and sPSGL-1 (B and C, cross).

The results of the preceding section indicate that 1) dimeric interactions required both receptor and ligand to be dimeric; 2) dimeric interactions formed more frequently than monomeric interactions; and 3) E-selectin had the same affinity with each leg of dimeric PSGL-1 as with monomeric sPSGL-1. We next compared the 2D binding of sPSGL-1 and PSGL-1 to E-selectin on the surface of two cell types: HAECs stimulated by IL-1β to induce native E-selectin expression at two levels (Fig. 3A and B) and CHO cells transfected to express recombinant E-selectin (Fig. 3C). Nonspecific adhesions were controlled by using an anti-E-selectin mAb ES1 and the calcium chelator EDTA to inhibit specific E-selectin binding. The adhesion frequency vs. contact duration curves of cell surface E-selectin (Fig. 3) showed similar kinetics as purified E-selectin-Ig coated on RBCs (Fig. 2C), imparting confidence to applying the premises established in the preceding section to analyze the data in this section.

Similar to Fig. 2C, binding of cell surface E-selectin to dimeric PSGL-1 was significantly higher than that to monomeric sPSGL-1 at all contact durations tested regardless of the site densities and cell types (Fig. 3). These data indicate that E-selectin was dimeric on the surface of HAEC and CHO cells because it supported dimeric interactions with PSGL-1 but monomeric interactions with sPSGL-1.

### Interactions of (s)PSGL-1 with L-selectin-Ig were Distinctly Different

**Figure 4:**
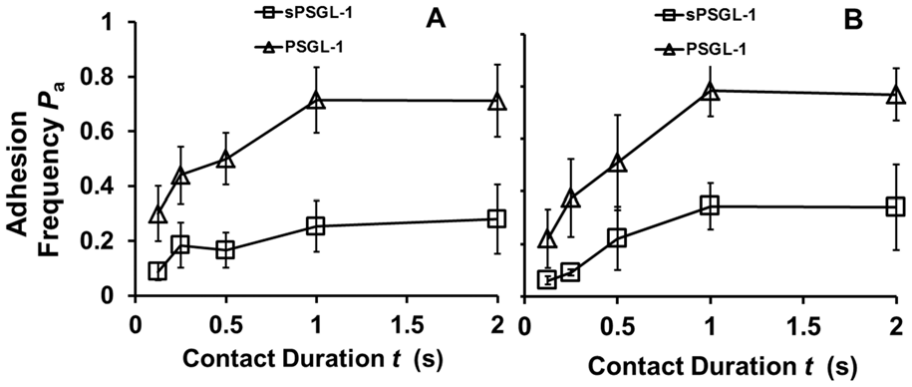
Binding curves of L-selectin-Ig interacting with (s)PSGL-1. Adhesion frequency vs. contact duration plots of RBCs coated with L-selectin-Ig at site density of 140 µm^−2^ (A) or 112 µm^−2^ (B) interacting with RBCs coated with matched densities of sPSGL-1 (square) or PSGL-1 (triangle).

We next employed the same strategy to analyze L-selectin. In contact durations from 0.125–2 s, RBCs bearing either of two site densities of dimeric L-selectin-Ig adhered to RBCs bearing dimeric PSGL-1 more frequently than to RBCs bearing monomeric sPSGL-1 (Fig. 4). This is similar to the binding curve pattern of E-selectin-Ig observed in Fig. 2C, confirming that dimeric vs. monomeric interactions of (s)PSGL-1 with E-selectin-Ig could be applied to the case of L-selectin-Ig. Thus, dimeric L-selectin-Ig formed dimeric bonds with dimeric PSGL-1 but monomeric bonds with monomeric sPSGL-1.

### Interactions of sPSGL-1 and PSGL-1 with Neutrophil L-selectin were Indistinguishable

**Figure 5:**
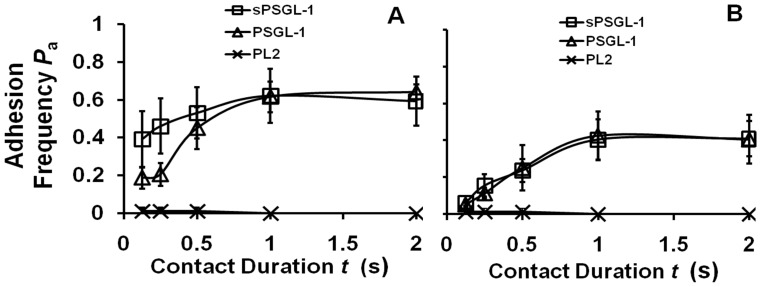
Binding curves of neutrophil L-selectin interacting with (s)PSGL-1. Adhesion frequency vs. contact duration plots of neutrophils constitutively expressing native L-selectin interacting with RBCs coated with matched densities of sPSGL-1 (square) or PSGL-1 (triangle) at 59 µm^−2^ (A) or 25 µm^−2^ (B). Control for nonspecific adhesion was measured using RBCs coated with the capture antibody PL2 without incubation with (s)PSGL-1. The differences in the first three data points in (A) between sPSGL-1 and PSGL-1 interacting with L-selectin are not significant as assessed by the Student-t test (*p* = 0.32, 0.15, and 0.75).

We lastly compared the 2D binding of sPSGL-1 and PSGL-1 at two matched site densities to L-selectin constitutively expressed on human neutrophils. Nonspecific adhesions were controlled by using RBCs precoated with an anti-PSGL-1 capturing mAb PL2 but not further incubated with either form of PSGL-1, which abolished binding (Fig. 5). In contrast to the pattern seen with Fig. 4, the binding curves of dimeric PSGL-1 and monomeric sPSGL-1 were indistinguishable. These results indicate that each of the two members of dimeric PSGL-1 interacted with L-selectin with the same binding affinity, similar to the case of sE-selectin (Fig. 2A and B). Like the sE-selectin data, the data in Fig. 5 also suggest that L-selectin was a monomer on the surface of neutrophils because it supported monomeric interactions with both monomeric sPSGL-1 and dimeric PSGL-1.

## Discussion

Using adhesion frequency measurements, we have demonstrated that E-selectin on endothelial cells or transfected CHO cells supports dimeric interactions with dimeric PSGL-1, whereas L-selectin on neutrophils supports only monomeric interactions with dimeric PSGL-1.

Our observed monomeric interactions of L-selectin with PSGL-1 are consistent with molecular stiffness measurements that L-selectin from human tonsils reconstituted into lipid bilayers forms monomeric bonds with PSGL-1 [14] and with biophysical measurements that L-selectin transmembrane and cytoplasmic domains do not dimerize in bacterial or synthetic cell membranes [13].

Our observed dimeric inteactions of E-selectin with PSGL-1 differ from previous observations that E-selectin from lysates of CHO cell transfectants reconstituted into supported lipid bilayers forms monomeric bonds with PSGL-1, although P-selectin from lysates of human platelets reconstituted in the same lipid bilayers forms dimeric bonds with PSGL-1 [14]. E-selectin oligomerization may be driven by weak noncovalent interactions that are disrupted after detergent solubilization of membranes. P-selectin dimerizes by noncovalent association of transmembrane domains (Ushiyama et al 1993). These associations may require its GxxxG sequence, a known dimerization motif [37], [38]. PSGL-1 dimerizes by noncovalent interactions between both transmembrane and cytoplasmic domains, which are stabilized by a disulfide bond formed immediately above the plasma membrane (Moore et al 1992, Epperson et al 2000, Miner et al 2011). Although the transmembrane domain of E-selectin does not contain a GxxxG sequence, our data suggest that close *cis* interactions of E-selectin in the membrane enable dimeric bonds with PSGL-1. How an E-selectin dimer is stabilized on a cell is an interesting question for future studies.

Many adhesion molecules are constitutively expressed on the cell surface as homodimers or oligomers. In addition to P-selectin and PSGL-1, the β_1_ [39], β_2_ [40], β_3_ [41]–[43], and α_IIb_ [44] integrin subunits can form clusters. Other examples include intercellular adhesion molecule 1 (ICAM-1) [45], platelet endothelial cell adhesion molecule-1 (PECAM-1) [46], and cadherins [47]. In addition, many cell surface receptors can be induced to form oligomers or increase the size of preformed oligomers as a result of intracellular signaling or ligand engagement [16]. Examples include T cell receptors [48], [49], B cell receptors [50], and Fc receptors [51], [52]. Importantly, in addition to strengthening receptor–ligand interactions, oligomeric binding plays a crucial role in many receptor-mediated signaling processes, making the analysis of oligomeric interaction a highly relevant area of study.

Receptor oligomerization is usually demonstrated by biochemical methods, e.g., chemical crosslinking, as in the case of α_IIb_β_3_ integrin [53]. Because ligand binding to adhesion receptors provides physical linkage to anchor the cell, oligomeric interactions can be analyzed by biomechanical assays. For example, dimeric interactions between P-selectin and PSGL-1 prolong bond lifetimes [10], increase molecular stiffness [14], and stabilize cell rolling in shear flow [9]. Multimeric bonds have also been characterized by increase in bond rupture forces [54]–[57]. The present work has demonstrated a new method of analyzing dimeric receptor-ligand binding by measuring 2D kinetics on the cell membrane. However, these methods also have limitations.

One limitation is the resolution of the our methods. The biomechanical assays based on pico-force techniques usually have sufficiently high resolutions for mechanical variables such as bond lifetime, molecular stiffness, cell rolling velocity, bond rupture force, and adhesion frequency. However, they must be combined with assays to measure site densities of the interacting molecules. Biochemical assays sometimes suffer from inter-experimental variations, as measurements depend on the activities of the reagents that can vary in each preparation. This may explain the apparent discrepancies between Fig. 2A and B, where the measured difference in steady-state adhesion levels is nearly two-fold smaller than that predicted from the 2.7-fold difference in the sE-selectin site densities. For this reason, quantitative comparison should be done in side-by-side experiments using the same preparation of reagents with proper controls to prevent inter-experimental variations from negating key conclusions. This precaution was strictly followed for the comparative data in each panel of Figs. 2–5, ensuring the reliability of our conclusions.

To support dimeric interactions with dimeric PSGL-1, endothelial cell E-selectin must form oligomers of at least two proteins. If E-selectin forms oligomers larger than dimers on the endothelial cell surface, our data cannot determine the actual size of the oligomers. This limitation can be overcome by using oligomeric ligands of larger sizes to dissect the size of E-selectin oligomers combined with mathematical modeling to relate the binding curve to 2D kinetics rates. With such extensions, the present method has the potential to analyze oligomeric cross-junctional molecular binding at the interface of two interacting cells and extract intrinsic kinetic coefficients for such multimeric interactions.

In addition to molecular self-association, selectins cluster in specific membrane domains through interactions with other cellular elements. L-selectin clusters in the tips of leukocyte microvilli, which enhances tethering under flow [58]. P- and E-selectin cluster in clathrin-coated pits and E-selectin clusters in lipid rafts of endothelial cells; these clusters promote slower and more stable leukocyte rolling [59]–[61]. Dimerization of P-selectin may cooperate with P-selectin clustering in clathrin-coated pits to optimize leukocyte rolling [59], [62]. For a given dimeric ligand, whether and how effectively it forms a dimeric bond with a dimeric receptor may depend on the on-rate and the separation distance between the two members of the dimeric receptor, as close proximity of the members may compensate for slow on-rate to promote a dimeric interaction. The on-rate (but not off-rate) of 2D receptor–ligand binding kinetics is impacted by the microtopology [63] and stiffness [64] of the cell surface presenting the receptor as well as the membrane anchor [20], length and orientation [65] of the receptor and/or ligand. These provide potential mechanisms for regulation of dimeric/oligomeric interactions. The biological significance of cells expressing E-selectin as dimers/oligomers but L-selectin as monomers is an important topic for future investigation.
